# Mesenteric gastrointestinal stromal tumour presenting as intracranial space occupying lesion

**DOI:** 10.1186/1477-7819-4-78

**Published:** 2006-11-14

**Authors:** Tarun Puri, Gowthaman Gunabushanam, Monica Malik, Shikha Goyal, Anup K Das, Pramod K Julka, Goura K Rath

**Affiliations:** 1 Department of Radiotherapy and Oncology, All India Institute of Medical Sciences, New Delhi, India; 2 Department of Radiodiagnosis, All India Institute of Medical Sciences, New Delhi, India; 3 Department of Pathology, All India Institute of Medical Sciences, New Delhi, India

## Abstract

**Background:**

Gastrointestinal stromal tumours (GIST) usually present with non-specific gastrointestinal symptoms such as abdominal mass, pain, anorexia and bowel obstruction.

**Methods:**

We report a case of a 42 year old male who presented with a solitary intracranial space occupying lesion which was established as a metastasis from a mesenteric tumour.

**Results:**

The patient was initially treated as a metastatic sarcoma, but a lack of response to chemotherapy prompted testing for CD117 which returned positive. A diagnosis of mesenteric GIST presenting as solitary brain metastasis was made, and the patient was treated with imatinib.

**Conclusion:**

We recommend that all sarcomas with either an intraabdominal or unknown origin be routinely tested for CD117 to rule out GIST.

## Background

Gastrointestinal stromal tumours (GIST) are thought to arise from the interstitial cells of Cajal, the intestinal pacemaker cells [1]. GIST commonly arises from the stomach and small intestine and usually presents with non-specific abdominal symptoms [2]. GIST mostly metastasizes within the abdomen [3]. To our knowledge, GIST presenting with brain metastasis has not been reported in the literature. We report a case of a 42 year old male who presented with a solitary intracranial space occupying lesion which was subsequently established to be a metastasis from a mesenteric GIST.

## Case presentation

**Figure 1:**
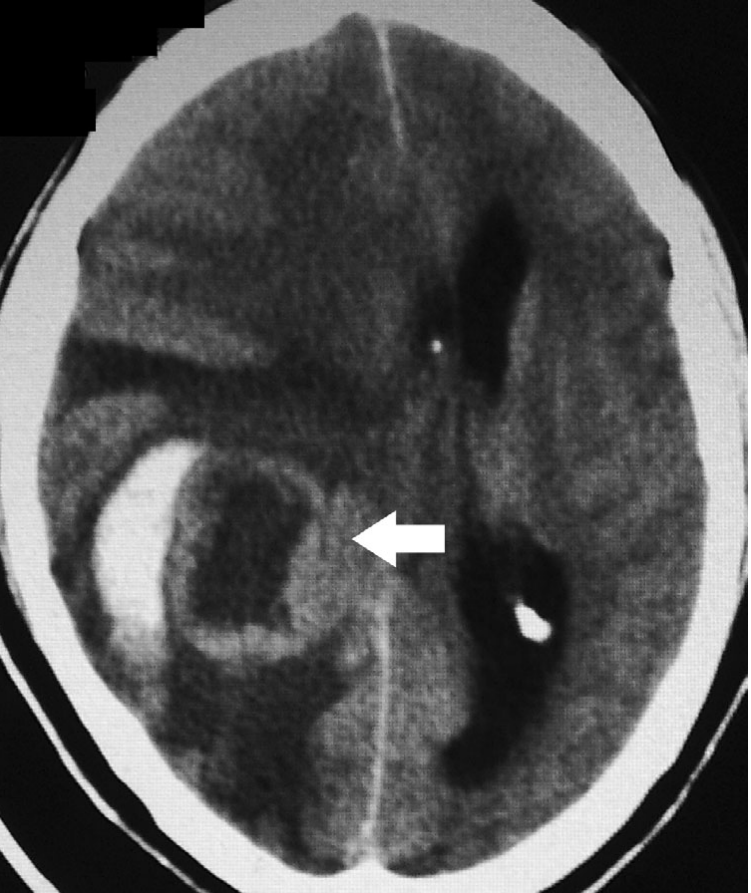
CT scan of the brain showing a 3.5 cm sized ring-enhancing mass (arrow) in the right parietal lobe with significant mass effect and compression of the right lateral ventricle. There is significant peri-lesional oedema.

**Figure 2:**
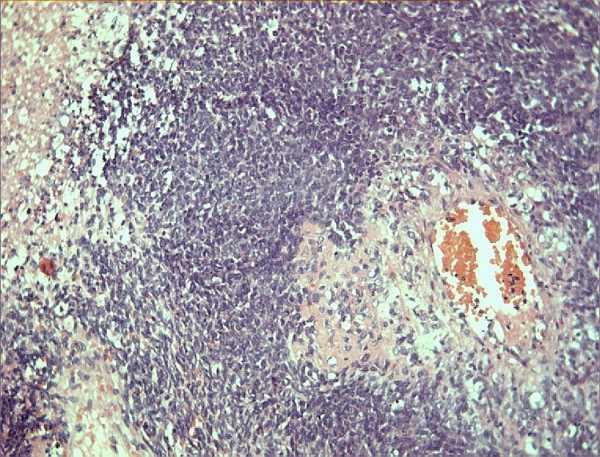
Section from operative specimen from right parietal lobe lesion shows features of undifferentiated sarcoma with areas of necrosis. (Haematoxylin & Eosin, 400×)

A 42-year-old male presented in January 2004 complaining of headache and left-sided weakness of eight months duration and vomiting of four months duration. There was no history of trauma, seizures or loss of consciousness. Computed Tomography (CT) scan of the brain (Figure 1) showed a 3.5 cm sized ring-enhancing mass in the right parietal lobe with significant perilesional oedema. Magnetic resonance imaging (MRI) of the head showed the mass to be isointense on T1 and hypointense on T2 weighted images. The patient underwent right parietal craniotomy with gross total excision of the tumour. Histopathology of the operative specimen showed features of undifferentiated sarcoma with areas of necrosis (Figure 2). The pathological differential diagnoses given were metastatic sarcoma and primary intracranial sarcoma. Radiographic skeletal survey, chest x-ray and ultrasonogram of the abdomen were normal at this time. The patient was treated with postoperative cranial radiotherapy to a dose of 60 Gray with concomitant chemotherapy with carboplatin at a dose of 150 mg weekly.

**Figure 3:**
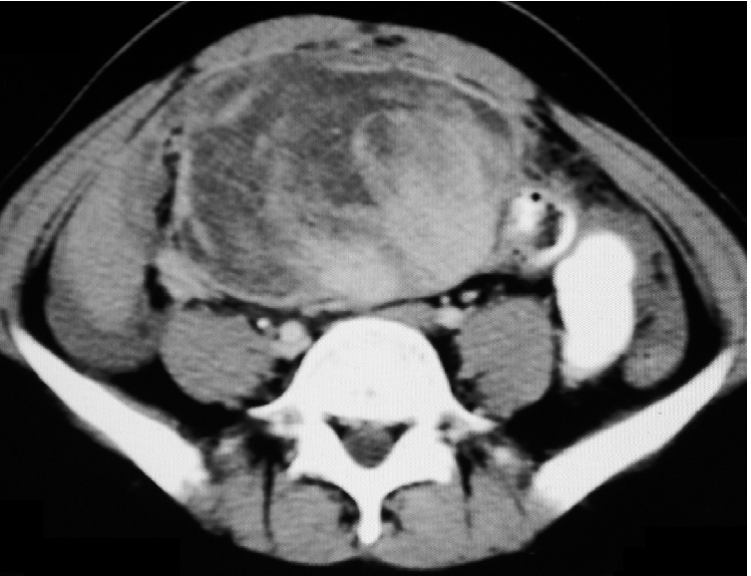
Contrast enhanced CT (CECT) scan of the abdomen done in November 2004 after 6 cycles of ifosfamide and epirubicin combination chemotherapy showing a 10 × 8 cm sized heterogeneously enhancing mesenteric mass.

The patient remained asymptomatic till June 2004 when he complained of abdominal fullness and pain. CT of the abdomen showed a large mesenteric mass measuring 8 × 6 cm in size. An ultrasound guided tru-cut biopsy from the mesenteric mass revealed a malignant spindle cell tumour with morphology similar to that of the brain tumour. The mitotic index of the abdominal tumour was 10 per 50 HPF (high power fields). Immunohistochemistry was positive for CD34, vimentin; and negative for GFAP (glial fibrillary acidic protein) and SMA (smooth muscle actin). Thereafter the patient was treated with six cycles of combination chemotherapy with ifosfamide 1.8 gm/m^2 ^i.v. D1-4 with mesna uroprotection and epirubicin 60 mg/m^2 ^i.v. in two divided doses, as 3-weekly cycles until November 2004. However, there was no apparent clinical response to chemotherapy, and abdomen CT (Figure 3) done at this time showed a 10 × 8 cm sized heterogeneously enhancing mesenteric mass.

**Figure 4:**
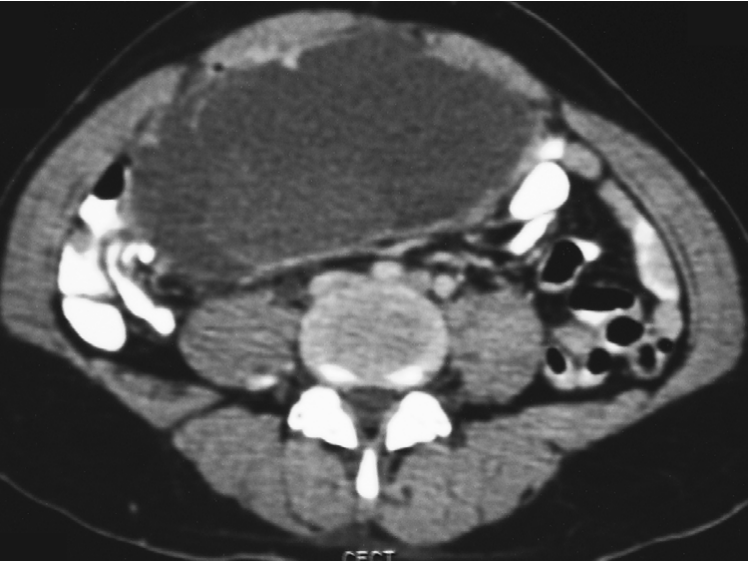
CECT of the abdomen done after three months of imatinib therapy showing a good response, evidenced by a cystic conversion of the mesenteric mass with no enhancing areas within.

**Figure 5:**
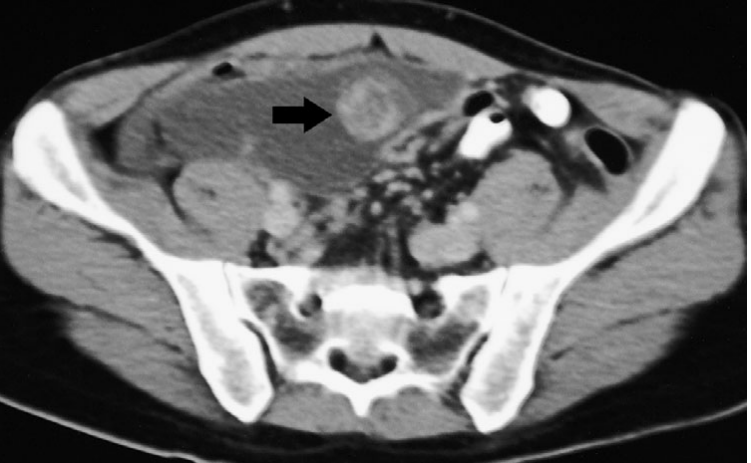
CECT of the abdomen done three months after that in Figure 4 showing a focal nodular area of enhancement (arrow) within the mesenteric mass, which is suggestive of disease progression. The patient died four months later.

Given the failed response to chemotherapy, the pathology specimen was retested for CD117/c-kit which turned out to be positive. Thus, the patient was diagnosed as a primary mesenteric gastrointestinal stromal tumour presenting as solitary brain metastasis. The patient was then treated with imatinib mesylate 600 mg oral daily. CT scan of the chest and abdomen after three months of therapy showed a good response, evidenced by a cystic conversion of tumour mass with no visible areas of enhancement (Figure 4). The patient continued to remain on imatinib therapy. However, after the initial response, CT scan of abdomen done after another three months showed a focal nodular area of enhancement (arrow) within the mesenteric mass, suggestive of disease progression. (Figure 5) The patient subsequently developed cachexia and died four months later.

## Discussion

Gastrointestinal stromal tumours (GIST) are thought to arise from the interstitial cells of Cajal, the intestinal pacemaker cells [1]. GIST most commonly arises from the stomach (60–70%) and small intestine (20–30%) with less than 10% arising from the rest of gastrointestinal tract (oesophagus, colon, rectum) or extraintestinal sites (omentum, mesentery, retroperitoneum) [2-4].

The clinical presentation of patients with GIST can vary depending on the tumour location, size and aggressiveness. Typically, patients present with non-specific gastrointestinal symptoms such as abdominal mass, pain, vomiting, anorexia and bowel obstruction. Rarely, patients may present with acute haemorrhage into peritoneal cavity from tumour rupture or cutaneous metastases [5,6].

GIST mostly metastasizes within the abdomen. In one series of 83 patients [7], the common sites of metastases were to the liver (46%) and peritoneum (41%). Other sites of metastases reported in this series include the retroperitoneum, lung, bone and abdominal scar. Involvement of the central nervous system (CNS) by metastatic GIST is extremely rare. A review of the literature reveals only three reports of CNS involvement by GIST. In all cases, patients had known metastatic disease elsewhere prior to the development of CNS metastases. In one case [8], a 60-year-old male with diagnosed metastatic GIST developed sudden unilateral blindness that was found to be caused by metastatic involvement of the cavernous sinus. In the second case [9], a 47-year-old male who was being treated with imatinib for metastatic GIST developed multiple cerebral relapses even while systemic disease appeared to be controlled. In the third case [10], a 75-year-old male with multiple liver and peritoneal metastases developed a single intracranial lesion. In the third patient, all lesions including the intracranial lesion showed a good response to imatinib therapy.

In the present case, the patient had an unusual presentation as an intracranial metastasis. The patient was initially treated as a metastatic sarcoma from unknown primary due to the absence of extracranial disease on radiological workup. Even after the detection of the mesenteric primary, the patient was initially treated as a mesenteric sarcoma as histopathology showed a sarcoma which was positive for CD34 and vimentin antigen. The possibility of GIST was suspected only after the patient failed to respond to ifosfamide and epirubicin combination chemotherapy. As the immunohistochemisty returned positive for CD117 at this stage, the patient was treated with imatinib [11,12]. A good initial treatment response was noted with imatinib, with a reduction in tumour size and the development of cystic changes in the tumour mass. However, the patient subsequently developed progressive disease and died.

## Conclusion

The present report describes a unique presentation of GIST as intracranial metastasis. To the best of our knowledge, this is the first report of its kind in the literature. The true nature of disease remained obscure for a considerable duration; this delay could be attributed, in part to the atypical presentation and to the lack of routine testing for CD117. We recommend that all cases of sarcomas with an intraabdominal origin or unknown origin be routinely tested for CD117 to detect GIST as these tumours usually respond to imatinib.

## Competing interests

The author(s) declare that they have no competing interests.

## Authors' contributions

TP, GG, MM, SG, AKD, PKJ, GKR contributed significantly to draft the manuscript. All authors read and approved the final manuscript.
